# Fast screening of gold in rock samples using polyurethane foam extraction and inductively coupled plasma mass spectrometry determination

**DOI:** 10.1016/j.mex.2025.103170

**Published:** 2025-01-14

**Authors:** Jalal Hassan, Naeemeh Zari, Mohammad-Hadi Karbasi

**Affiliations:** a Division of Toxicology, Department of Comparative Bioscience, Faculty of Veterinary Medicine, University of Tehran, Tehran, Iran; b Geology Survey of Iran, Ministry of Industry and Mineral, Karaj, Iran; c Iranian Mineral Processing Research Center, Ministry of Industry and Mineral, Karaj, Iran

**Keywords:** Gold, Foam, ICP/MS, soil, Rapid test

## Abstract

In this work, the volume of sample solution and concentration of gold was optimized for extraction with foam in dimensions (1 × 1 × 1) and then was used for extraction from soil samples. The results showed that the proposed technique has a good analytical efficiency compared to the standard fire assay method and the accuracy of work is in the range of 74–125 %. The equation of linear calibration curve was obtained with regression coefficient better than 0.9997, and the detection and quantification limit of the gold in aqueous and soil sample obtained 0.25 and 0.8 µg kg ^−1^, respectively.

• This method is inexpensive and fast for determination of gold in various samples.
• This method has high thought of sample determination.
• This method is green chemistry method.

## Graphical abstract

Aqua regia digestion, PUF extraction and ICP-MS determination for rapid separation, pre-concentration and determination for ultra trace amount of gold.

**Figure:**
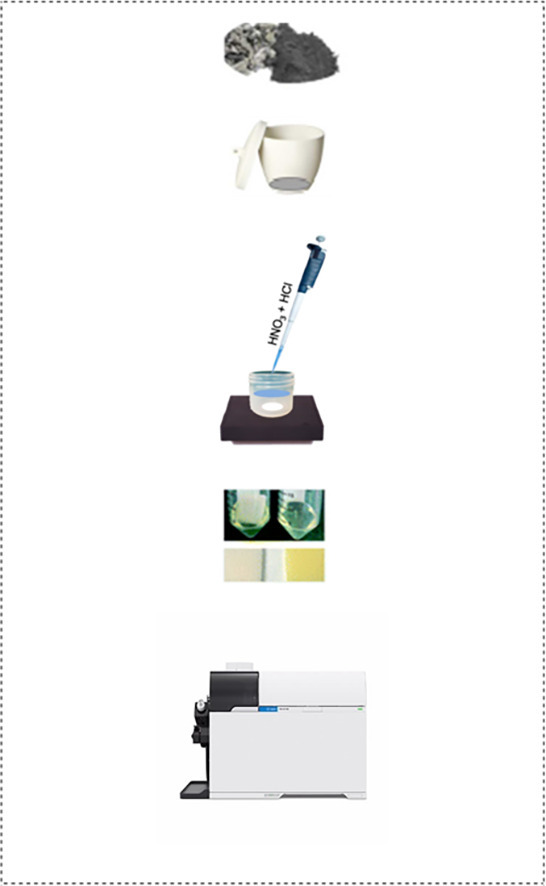

Specifications table

**Table:** 

Subject area:	Chemistry
More specific subject area:	*Analysis*
Name of your method:	Rapid test
Name and reference of original method:	*ASTM*
Resource availability:	*No*

## Background

The purpose of this work is to develop a fast, cheap and available method for monitoring gold in various exploratory samples. In this study, polyurethane foam is used to determine gold in one grams of rock powder. To obtain high gold recovery, the experimental conditions for adsorption and elution are optimized. Ten reference materials (RM) from different matrices have been used to evaluate the method and this method has been compared with fire assay method.

## Method details

Gold belongs to the group of elements which occur on the earth with very low natural abundance. The amount of gold is in the range of 1–4 ng g ^−1^ in rocks and soils, 0.05–0.2 ng mL^−1^ in seawater and river water samples, respectively [1]. Therefore, cost-effective, simple, and selective methods for pre-concentration and determination of traces of gold are of great importance in routine analysis. Measuring and analyzing gold has always been one of the most important challenges in the exploration and determination stages of gold deposit due to the complexity of the problem and its very small quantity [2]. Rapid, sensitive and accurate measurement of gold is a long-term concern in earth sciences, mineral industries and analytical methods. Due to their simplicity, economy and availability in various institutions, inductivity coupled plasma mass spectrometry (ICP-MS) and graphite furnace atomic absorption spectroscopy (GFAAS) have become the main tools for measuring trace amount of gold and some of trace elements in real samples [[3], [4], [5]]. Different methods of preparation and measurement of gold in soil samples can be found in different sources. For example, fire assay technique is the oldest and safest preparation method for analyzing gold in ores and has remained the industry standard method to this day. Briefly in lead fire assay procedure, gold is collected in a lead button while the rest of the components enter the slag. Lead button is processed and removed by adsorption on bone ash or magnetized cupels, leaving the precious metals behind as a metallic bead (prill). The peril is digested in aqua regia and the concentration of gold is determined by spectroscopic methods such as atomic absorption spectroscopy (AAS), inductivity coupled optical emission spectrometry (ICP-OES), and ICP-MS. This method has some disadvantages such as time consuming, costly and polluting the environment [6]. Due to challenges from sample (low content of analyte, chemical interferences,) and instruments (spectral interferences, sensitivity…) together, different pretreatment methods for enrichment and separation of gold from the matrix have been continuously studied and reported. One of these extraction methods is the use of polyurethane foam, which researchers have used in various ways [[7], [8], [9], [10], [11]].

## Experimental

### Apparatus

Fire-resistant crucibles of 150 mL, iron cups, couples 60 mm, shaft furnace with an operating temperature of 1070–1100 °C, cupellation furnace of 20 kW. A Model 220Z Graphite furnace atomic absorption spectrometer equipped with Zeeman background (Varian, Australia) and pyrolytic partitioned graphite tubes (Varian, Australia) were used. Measurements were carried out using our previous work [8]. Gold was analyzed by inductively coupled plasma mass spectrometer (ICP-MS) type Agilent 7900 equipped with a Pelletier-cooled, inert sample introduction system. All parts of ICP-MS were purchased from Agilent Technologies and to reduce the high memory effect of Au, 10 % hydrochloric acid was used as a cleaning solution. The operating conditions for the ICPMS are given in Table 1.

**Table 1:** Operating conditions and methodical set-up of ICPMS.

Parameter	Value
RF power (w)	1550
Cool gas flow (L/min)	15
Auxiliary gas flow (L/min)	0.9
Sampling gas flow (L/min)	1
Au isotope (m*/z*)	197
Speed of nebulizer pump(rpm)	0.3

### Materials

All reagents were of analytical grade, unless otherwise stated. PbO, Na_2_CO_3_, Na_2_B_4_O_7_, flour and iron (Flux for smelting), HF; HNO3, HCl, standard gold solutions were purchased from Merck company (Darmstadt, Germany). A stock solution of Au (1000 ppm) was prepared Merck company (Darmstadt, Germany). Solutions for the establishment of the calibration curve for gold were always prepared freshly on the day when recording the determination was to be performed. Commercial polyurethane foam was prepared from the shop (Tehran. Iran) and cut into cubic centimeters.

### Fire assay procedure

Each rock sample was grinded, homogenized and quartered. A representative sample was then pulverized to ∼200 mesh scale. The selection of the optimal sample weight was preceded by a trial of the acidic and for the combination of fire assay and AAS, a sample weight of 10 g was selected. Two procedures were applied to remove sulfur, which affects the loss of precious metals in the smelting process. In the first procedure samples were roasted at a temperature of 600 °C for duration of two hours and in the second iron was added to smelting charge. The flux components were as the following: lead oxide (20 g), sodium carbonate (25 g), sodium tetraborate (15 g) and reducing reagent flour (5 g). The charges prepared in such a manner that the flux was previously well mixed with the samples and quantitatively transferred to fire-resistant crucibles. The fusion process was performed at the temperature of 1070 °C for duration of 1 hr. The smelted material was poured into iron cups. After cooling, the lead with precious metals was separated from the slag by forging, placed into heated cupels and cupellated at a temperature between 890 and 940 °C in duration of 20–30 min. The remained bead (prill) retained at the bottom of the couple was dissolved with acids in the next procedure. The obtained silver and gold alloy bead, was dissolved with 2 mL of 50 % (v/v) nitric acid and then with 2 mL of aqua regia. To obtain silver in the form of complex compounds AgCl_2_^−^ and AgCl_3_^2−^ the solution was treated with 25 % (v/v) hydrochloric acid in a 100 mL volumetric flask. A sample blank was prepared in the same manner and with the same quantity of reagents, only without the analyzed sample.

### Extracting of gold from aqueous solution

Fifty mL aqueous sample in 10 % hydrochloric acid medium in a 50 mL falcon tube with polyurethane foam with dimensions (1 × 1 × 1) was extracted overnight (12 h). After extraction, the foam was rinsed, squeezed and then extracted with 20 mL of 1 % thiourea solution in a boiling water bath (100 °C) for 40 min. The foam was not rinsed after extraction but just squeezed and the amount of gold was determined by ICP-MS.

### Extracting of gold from soil

Each rock sample was grinded, homogenized and quartered. A representative sample was then pulverized to ∼200 mesh scale. The samples were dried overnight at 80 °C. 10.0 g of each sample was weighed and digested with 20 mL of concentrated aqua regia by stirring on a hot plate at 80 °C and heated to half its volume. After cooling, the solution of each sample was centrifuged and filtered. The filtered solution was diluted with distilled water in 50.0 mL volumetric flask. This sample was extracted with polyurethane foam with dimensions (2 × 2 × 2) for overnight. The foams were squeezed after extraction and then extracted with 20 mL of 1 % thiourea solution in a boiling water bath (100 °C) for 60 min.

## Method validation

**Fig. 1:**
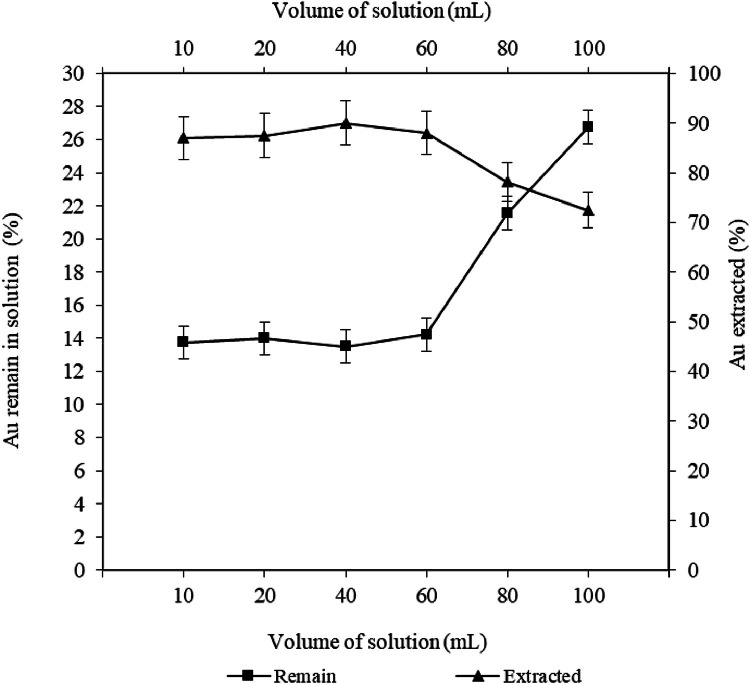
Effect of breakthrough volume on extraction of gold from aqueous solution by.

The breakthrough volume:

A 500 mL of solution containing 40 µgL^−1^ of gold was prepared in 10 % hydrochloric and was confirmed by ICPMS. Then, amounts of 10, 20, 40, 60, 80, 100 mL of gold solution were extracted from a 40 µgL^−1^ sample in 10 % hydrochloric acid medium with polyurethane foam (1 × 1 × 1) overnight (12 h). The foams were squeezed after extraction and then extracted with 20 mL of 1 % thiourea solution in a boiling water bath (100 °C) for 20 min. The foam was not rinsed after extraction, only squeezed and the amounts of unabsorbed and extracted gold were measured Fig. 1.

As can be seen, for a solution with a concentration of 40 micrograms per liter (µgL^−1^) up to a volume of 60 mL, the extraction efficiency and the amount of gold remaining in the solution is in the range of 87–90 and 13–14 %, respectively. Therefore, the volume of 50 mL solution was selected for extraction in the next steps.

## Extracting of gold from aqueous solution

Fifty mL of 0.5, 1, 3, 5, 7, 10 and 40 µgL^−1^ samples in 10 % hydrochloric acid medium in a 50 mL falcon tube with polyurethane foam with dimensions (1 × 1 × 1) was extracted overnight (12 h). After extraction, the foam was rinsed, squeezed and then extracted with 20 mL of 1 % thiourea solution in a boiling water bath (100 °C) for twenty minutes. The foam was not rinsed after extraction but just squeezed and the amount of gold was measured by furnace atomic absorption (Table 2).

**Table 2:** Recovery of gold from 50 mL of spiked solution into 20 mL extraction solution by foam extraction method.

C_i_ (µgL^−1^)	C_f_ (µgL^−1^)	Recovery ± Sd (%)
0	0	0.0
0.50	1.4	112.0 ± 8
1.0	2.86	114.4 ± 6
3.0	6.9	92.0 ± 5
5.0	11.9	95.2 ± 5
7.0	16	91.4 ± 4
10.0	22	88.0 ± 7
40.0	100	100.0 ± 3

C_i_ = Concentration of gold in spiked solution, C_f_ = Concentration of gold in extraction solution.

As can be seen, the extraction efficiency in the concentration range of 0.50–40.0 micrograms per liter (µgL^−1^) is in the range of 88–114 %.

Analytical performance:

Under the optimal conditions, the calibration curve was drawn in the range of (0.8, 5, 10, 20, 40, 60, 80, 100) micrograms per liter (µgL^−1^) and the competency criteria of the method were calculated. The accuracy of the method was evaluated by analyzing standard soil samples. The equation of linear calibration curve was obtained as *Y* = 10323X with regression coefficient better than 0.9997, where Y is the signal measured by ICP-MS in the units of counts per second and gold concentration (X) is expressed in micrograms per liter (µgL^−1^). The detection and quantification limit of the gold in aqueous and soil sample, according to the criteria of 3S/m and 10S/m (where S is the standard deviation of the blank (5 repetitions) and m is the slope of the calibration diagram) obtained 0.25 and 0.80 µgkg ^−1^, respectively. As can be seen in the Table 3, the percentage of gold recovery is in the range of 74–125 % and by comparing the results obtained in this study and the true value using paired *t*-test, it was found that there is no significant difference between them. As can be seen, there is no significant difference between the two measurements methods used in this paper, but the measurement method with foam is much cheaper and has a higher automation capability than fire assay method.

**Table 3:** Measurement of gold in standard soil samples by the presented method and fire assay method.

Code	C (ng g^−1^)			
	Obtained	Fire assay	Certified	Recovery %
GD50257	260	310	316	82
GD50299	5	3	4	125
GD51412	246	332	330	75
GD52227	23	19	20	115
GD52761	23	22	24	95
GD10136	470	540	470	100
GD10276	855	1210	1030	83
GD10738	2600	2790	2830	92
GD10861	730	860	760	96
GD11126	1050	1220	1300	81
GD11965	320	430	310	103
GD12310	1050	1450	1360	77
GD12280	625	900	850	74
GD12578	1290	1660	1580	82

We also measured gold in >100 real samples with each method and did not see any significant differences in the obtained results (results are not given).

## Conclusion

The proposed method is cheap, fast and has the ability to automate the measurement of gold in the rock. In this method, one gram of rock is used and at the same time, a large number of samples can be prepared and extracted with cheap polyurethane foam and then measured using an ICP/MS device.

## Limitations

Not applicable.

## Ethics statement

This study, titled “Fast screening of gold in rock samples using polyurethane foam extraction inductively coupled plasma mass spectrometry determination” was conducted in strict accordance with the ethical guidelines of the Methods X Journal.

## CRediT author statement

**Jalal Hassan:** Methodology. **Naeemeh Zari:** Analysis Data acquisition, Writing. **Mohammad-Hadi Karbasi:** Methodology. **Jalal Hassan:** Writing review and editing Data acquisition.

## Data Availability

Data will be made available on request.
